# The use of continuous electronic prescribing data to infer trends in antimicrobial consumption and estimate the impact of stewardship interventions in hospitalized children

**DOI:** 10.1093/jac/dkab187

**Published:** 2021-06-10

**Authors:** S. Channon-Wells, M. Kwok, J. Booth, A. Bamford, P. Konstanty, J. Hatcher, G. Dixon, P. J. Diggle, J. F. Standing, A. D. Irwin

**Affiliations:** 1 Oxford University Hospitals NHS Foundation Trust, Oxford, UK; 2 UCL School of Pharmacy, University College London, London, UK; 3 Great Ormond Street Hospital for Children NHS Foundation Trust, London, UK; 4 UCL Great Ormond Street Institute of Child Health, London, UK; 5 CHICAS, Lancaster Medical School, Lancaster University, Bailrigg, Lancaster, UK; 6 Infection Management and Prevention Service, Queensland Children’s Hospital, Brisbane, Queensland, Australia; 7 UQ Centre for Clinical Research, The University of Queensland, Brisbane, Queensland, Australia

## Abstract

**Background:**

Understanding antimicrobial consumption is essential to mitigate the development of antimicrobial resistance, yet robust data in children are sparse and methodologically limited. Electronic prescribing systems provide an important opportunity to analyse and report antimicrobial consumption in detail.

**Objectives:**

We investigated the value of electronic prescribing data from a tertiary children’s hospital to report temporal trends in antimicrobial consumption in hospitalized children and compare commonly used metrics of antimicrobial consumption.

**Methods:**

Daily measures of antimicrobial consumption [days of therapy (DOT) and DDDs] were derived from the electronic prescribing system between 2010 and 2018. Autoregressive moving-average models were used to infer trends and the estimates were compared with simulated point prevalence surveys (PPSs).

**Results:**

More than 1.3 million antimicrobial administrations were analysed. There was significant daily and seasonal variation in overall consumption, which reduced annually by 1.77% (95% CI 0.50% to 3.02%). Relative consumption of meropenem decreased by 6.6% annually (95% CI −3.5% to 15.8%) following the expansion of the hospital antimicrobial stewardship programme. DOT and DDDs exhibited similar trends for most antimicrobials, though inconsistencies were observed where changes to dosage guidelines altered consumption calculation by DDDs, but not DOT. PPS simulations resulted in estimates of change over time, which converged on the model estimates, but with much less precision.

**Conclusions:**

Electronic prescribing systems offer significant opportunities to better understand and report antimicrobial consumption in children. This approach to modelling administration data overcomes the limitations of using interval data and dispensary data. It provides substantially more detailed inferences on prescribing patterns and the potential impact of stewardship interventions.

## Introduction

Antimicrobial resistance (AMR) represents a significant and growing threat to public health. In response, national and international bodies have developed strategic plans to mitigate its effects. A central element of these is the strengthening of capacity to carry out surveillance of both antimicrobial consumption and resistance.^1^^,^^2^ In 2017, The National Health Service England (NHSE) Commissioning for Quality and Innovation for Antimicrobial Resistance (CQUIN AMR) guidance tasked institutions with reducing total antimicrobial prescribing. Additionally, the CQUIN asked NHS hospitals to reduce prescribing of piperacillin/tazobactam and carbapenems by 1%–2%, with targets based on previous local performance.

The methods used to measure and report antimicrobial consumption in children are limited and consequently few systematic data are available. International surveillance systems such as the ECDC Annual Epidemiological Report make little or no reference to consumption in children.^2^ Those studies quantifying consumption in hospitalized children have either used the imperfect DDD metric, derived from pharmacy dispensary data, or depended upon a point prevalence survey (PPS) methodology.^3^ The Antibiotic Resistance and Prescribing in European Children network (ARPEC, now Global-ARPEC) has successfully brought together hundreds of contributing centres to provide an international snapshot of antimicrobial consumption in children.^4^ While these data have been highly informative in providing a high-level indication of antimicrobial consumption in children, the PPS methodology has limitations and makes the analysis of temporal trends challenging. The high staff cost associated with manual data collection is a further limitation.

Similarly, despite its widespread use, the DDD—defined as the ‘assumed average maintenance dose per day for a drug used for its main indication in adults’—is methodologically inadequate for paediatric populations, primarily due to dose variations by weight and age. Demographic differences between hospitals further limit its use in benchmarking. For paediatric hospitals, therefore, the metric ‘day(s) on therapy’ (DOT) combined with a suitable denominator, such as patient-days, appears to be a superior alternative.^5^ One DOT is counted for each antimicrobial agent administered on a calendar day, regardless of the number of administrations.

The increasingly widespread implementation of electronic health systems presents a valuable opportunity to improve the surveillance of antimicrobial consumption in children. Analysis of continuous administration data allows detailed inferences to be made across populations and over time, and will help to measure the impact of antimicrobial stewardship programmes (ASPs).^6^ Great Ormond Street Hospital for Children (GOSH) is a large, standalone children’s hospital in London, UK, providing tertiary and quaternary services. An electronic prescribing system was implemented in 2009, though its introduction into paediatric ICU (PICU) only occurred in 2016.

This study aimed to model antimicrobial consumption in children admitted to GOSH in order to observe seasonal and temporal variation, compare DOT with DDDs, compare the utility of continuous estimates with the established PPS methodology and report prescribing quality estimates using the recently published England-adapted ‘Access, Watch and Reserve’ (AWaRe) index.^7^ The AWaRe index categorizes antibiotics depending on their risk of toxicity and AMR into Access, Watch and Reserve groups and has been adapted to UK prescribing patterns from the WHO classification.^8^

## Methods

### Ethics

The study was approved by the Joint Research and Development Office of Great Ormond Street Hospital for Children NHS Foundation Trust and University College London, GOSH Institute of Child Health (UCL GOSH ICH; approval number 16IR34). Ethical approval for the retrospective analysis of de-identified data was obtained as part of the GOSH Clinical Informatics Data Store project (17/LO/0008).

### Setting

GOSH is a large standalone children’s hospital providing tertiary and quaternary services to children in the south-east of England, and supra-regional services to children throughout the UK and internationally. An extensive range of medical and surgical paediatric subspecialty services are provided, including neonatal, cardiac and PICU, solid organ transplant, haematology, oncology and HSCT. Data on all non-PICU antimicrobial administrations between 1 January 2010 and 31 December 2018 were retrieved from the electronic prescribing system of GOSH. Owing to a later implementation of electronic prescribing in PICU, PICU data were available from 1 January 2016. In response to a CQUIN target, GOSH strengthened its antimicrobial stewardship (AMS) service by funding additional consultant time for AMS, developing a ‘handshake’ stewardship strategy,^9^ which incorporated a new digital clinical decision support system for AMS rounds, enhancing education and awareness efforts and strengthening national and international networking. These interventions were implemented in late 2016.

### Prescribing data

All data relating to systemic antibacterial administrations were retrieved directly from two electronic medicines records systems: PICU data from CareVue (MedSphere Systems, UT, USA); and non-PICU data from JAC (JAC Computer Systems Ltd, Basildon, UK). Included antibacterials are shown in Table S1, available as Supplementary data at *JAC* Online. Individual antibacterial administrations were transformed into daily measures of consumption. We calculated DDDs based on the 2017 Anatomical Therapeutic Chemical (ATC) classification.^10^ DDDs were summed for medications with different DDD values according to the route of administration. Administration of any antibacterial on a calendar day, regardless of the number of administrations, constituted one DOT. Daily data on patient-days were used as a denominator. We estimated the quality of antimicrobial prescribing over time by reporting antimicrobial consumption by major subgroup of ATC group J01 (systemic antibacterials) and according to the England-adapted AWaRe index. For ease of illustration, these data were aggregated by month.

### Statistical methods

All analyses were undertaken in R,^11^ version 3.5.0, using the online GOSH Digital Research Environment (DRE). The DRE was established to enhance the secondary use of clinical data for research, by automatically uploading anonymized data to a cloud-based workspace. A detailed statistical methodology is reported in the Supplementary methods.

### Time-series analysis of antimicrobial consumption

Scatter plots and generalized additive model (GAM) plots of consumption over time were inspected to look for evidence of non-linearity on the log-odds scale.^12^ DOT and DDDs were modelled over time using an autoregressive moving-average model (AR-MA), with AR order 1 and MA order 1. Sine/cosine variables were included to represent seasonality. Polynomial terms were tested in the regression models where GAM plots suggested a non-linear relationship, and were retained where their inclusion improved the fit of the model to the data. Akaike information criterion (AIC) was used to compare model goodness of fit where appropriate. The time series of residuals was examined for evidence of autocorrelation. We tested the impact of an expansion of AMS capacity on meropenem and piperacillin/tazobactam consumption using a piecewise AR-MA model with the breakpoint specified by the date of the intervention.

### Simulation of PPSs

Traditional PPS estimates of antimicrobial use were simulated for meropenem by randomly sampling from 1 day 1 year in advance, with discrete uniform random noise added in (range ±4 days), for each year. We excluded weekends, to reflect usual PPS practice. We then calculated an estimated yearly change in antimicrobial use based on the DOT data available for these days, using a Poisson log-linear model. We also performed sensitivity analyses, including weekend sampling days, and altering the noise.

## Results

Excluding PICU patients, there were 1 343 748 antibacterial administrations in children admitted to GOSH between 1 January 2010 and 31 December 2018. The 10 most frequently administered antibacterials are shown in Table 1.

**Table 1.** Most frequently administered antibiotics

Antimicrobial	Administrations (count)
Piperacillin/tazobactam	226 316
Co-amoxiclav	152 180
Ciprofloxacin	122 385
Meropenem	102 590
Flucloxacillin	92 575
Amikacin	90 063
Vancomycin	75 803
Co-trimoxazole	64 404
Metronidazole	50 899
Ceftazidime	45 069

### Overall antibacterial consumption

**Figure 1.**
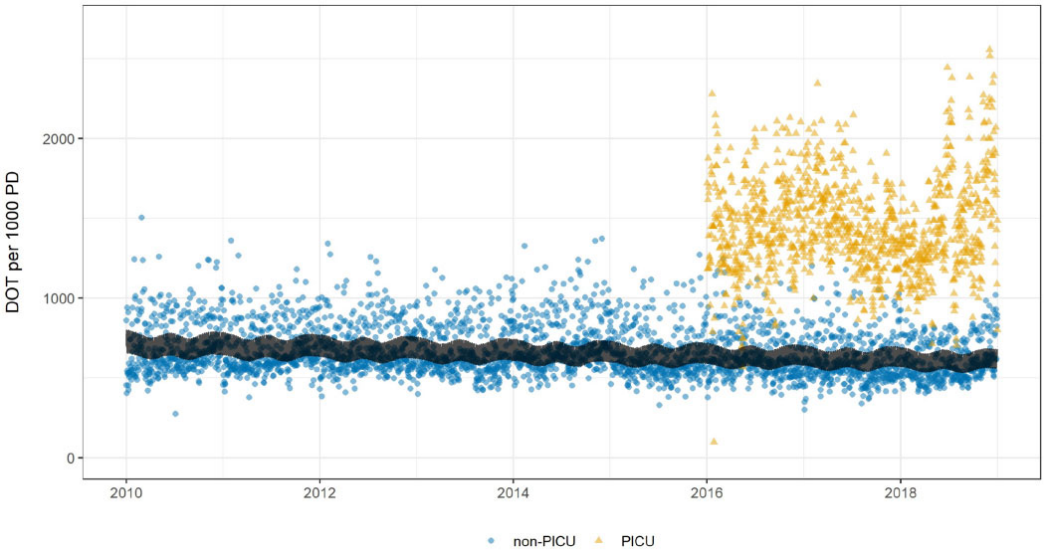
Daily consumption of all antimicrobials [DOT per 1000 patient-days (PD)]. Consumption in PICU from 2016 is illustrated by yellow triangles and non-PICU consumption by blue circles. The black line indicates fitted values from the AR-MA time-series model. This figure appears in colour in the online version of *JAC* and in black and white in the print version of *JAC*.

There was substantial day-to-day variation in antibacterial consumption, which ranged from 273 to 1504 DOT per 1000 patient-days. Median overall non-PICU consumption was 617 DOT per 1000 patient-days (IQR 551–746). Overall consumption in PICU patients was consistently higher, with median consumption of 1413 DOT per 1000 patient-days (IQR 1244–1629). Antibacterial consumption was higher at the weekend than the working week, as shown in Figures S1 and S2; median weekend DOT per 1000 patient-days was 757 (IQR 624–835), compared with a weekday median of 597 (IQR 546–659). Consumption was highly seasonal and there was an overall decline over time equating to an annual reduction of 1.77% (95% CI 0.50% to 3.02%). Daily consumption of all antibacterials is illustrated in Figure 1, alongside the fitted model. Residuals showed minor positive serial correlation. Model output is reported in Section 2.2 of the Supplementary methods.

### Consumption of specific antibacterials

**Figure 2.**
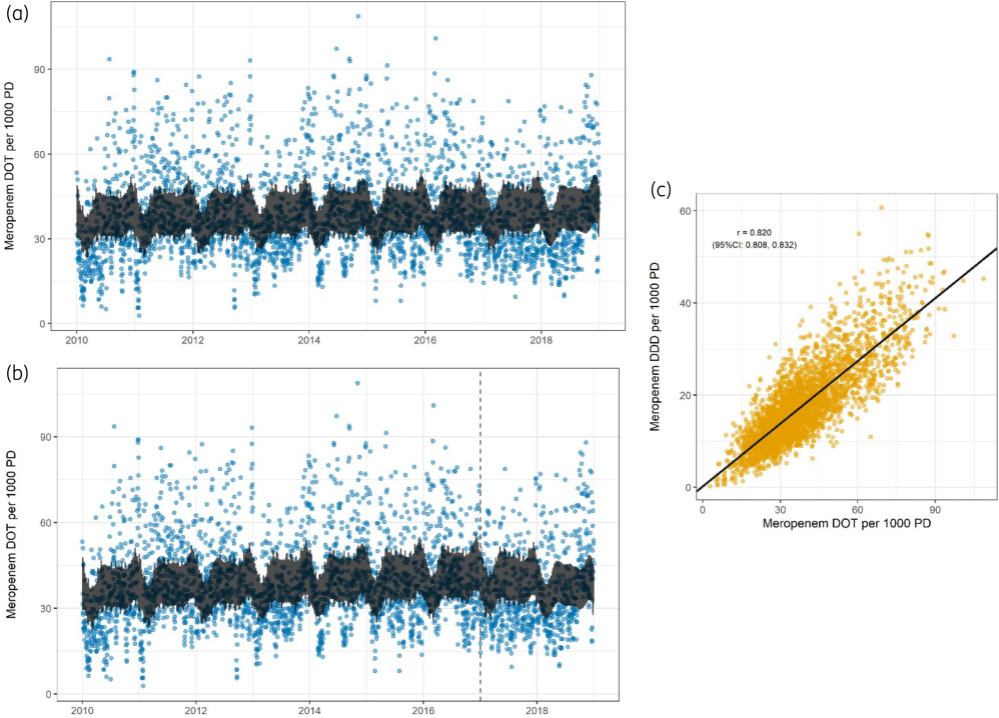
(a) Meropenem DOT per 1000 patient-days (PD); fitted AR-MA model. (b) Meropenem DOT per 1000 PD; fitted piecewise AR-MA model, breakpoint at start of expansion of AMS services at the end of 2016. (c) Meropenem DOT per 1000 PD against DDDs per 1000 PD, r = Pearson correlation coefficient with 95% CIs; linear regression line in black. This figure appears in colour in the online version of *JAC* and in black and white in the print version of *JAC*.

We performed time-series analysis of specific antibacterials, with a particular focus on meropenem and piperacillin/tazobactam. Weekly and seasonal variation was observed in meropenem consumption. Over time, there was an observed increase in the use of meropenem, equating to an annual increase in DOT of 0.45% (95% CI −1.48% to 2.43%). Piperacillin/tazobactam consumption also varied during the week and exhibited seasonality. Over time, there was a decrease in the use of piperacillin/tazobactam, with an annual decrease of 1.21% (95% CI −0.30% to 2.69%). Consumption of meropenem and piperacillin/tazobactam, expressed as DOT and DDDs, were highly correlated and produced similar estimates of change in consumption over time (Table S1). Figure 2 illustrates the fitted time-series model of meropenem consumption quantified in DOT (Figure 2a) and the correlation between DOT and DDDs (Figure 2c). Similar figures for piperacillin/tazobactam are available in Figure S3.

Agreement in consumption quantified by DDDs and DOT was not universally observed in all antibacterials, however. There was a small decrease in ciprofloxacin consumption quantified by DOT (−0.63%: 95% CI −2.36% to 1.14%) but an estimated 2.43% annual increase in DDDs observed (95% CI 0.54% to 4.35%). This discordance can be explained by an increase in the average dose of ciprofloxacin administered in early 2015 (Figure S4). Estimates of the annual change in consumption of individual antibacterials quantified by DOT and DDDs are compared in Table S1.

### Measures of quality of antimicrobial prescribing

**Figure 3.**
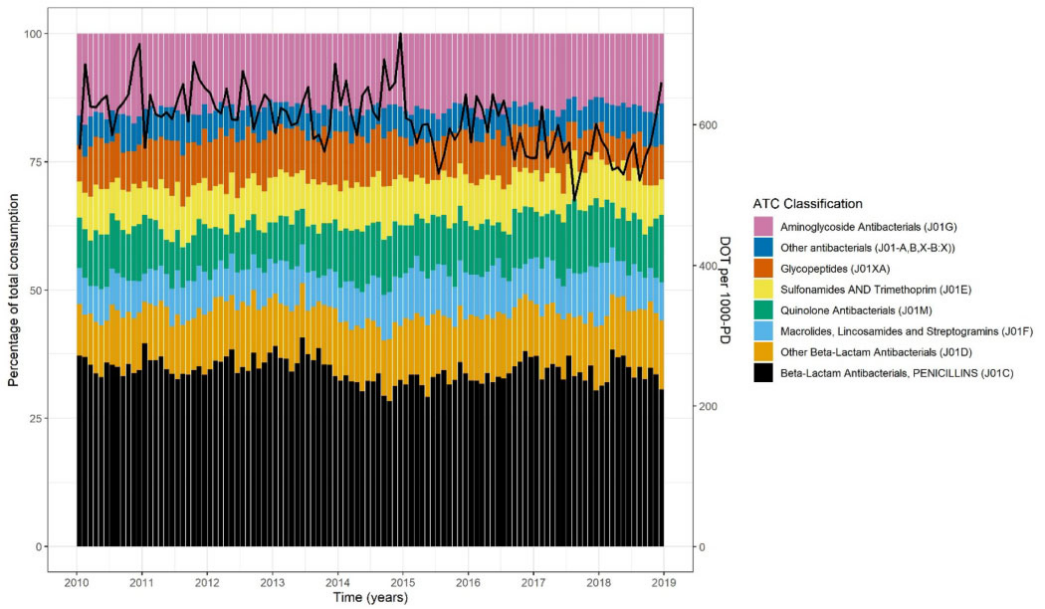
Percentage of total monthly consumption of antibacterial agents by ATC group. Black line indicates total monthly DOT per 1000 patient-days (PD). This figure appears in colour in the online version of *JAC* and in black and white in the print version of *JAC*.

**Figure 4.**
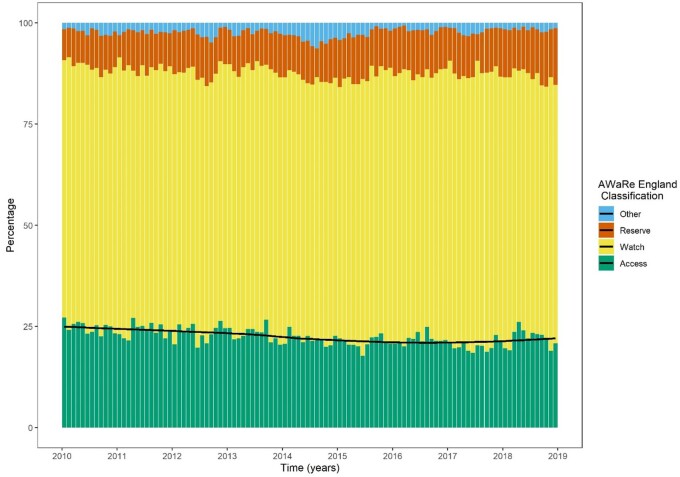
Monthly consumption of antibacterial agents by AWaRe England classification. The black line represents the trend (loess-fitted smoother) in monthly percentage of antimicrobials from the Access group. This figure appears in colour in the online version of *JAC* and in black and white in the print version of *JAC*.

Recently published quality measures of antimicrobial consumption are illustrated by grouping antibacterials by ATC major subgroup (Figure 3) and by the England-adapted AWaRe index classification (Figure 4) over time. Daily measures of consumption were aggregated into monthly interval data for ease of illustration. The percentage of antibiotic DOT from the lowest-risk Access group, as a proportion of all antibiotic use, decreased over time, representing just 22.5% of DOT over the study period (Figure 4, Table S2).

### Simulation of PPS data

**Figure 5.**
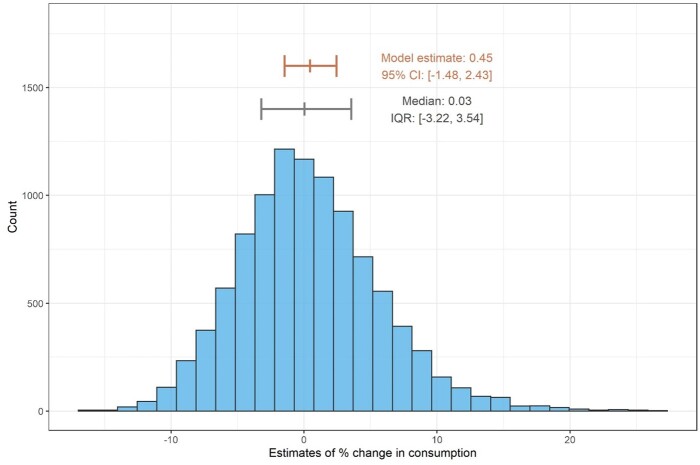
Histogram of the estimated annual change in meropenem consumption from 10 000 simulations of annual PPS data of meropenem consumption. The red line indicates the estimate of the change provided by the continuous model with 95% CIs. This figure appears in colour in the online version of *JAC* and in black and white in the print version of *JAC*.

We performed 10 000 simulations of annual PPS data for meropenem consumption. The estimates were neither precise nor accurate compared with the estimates of annual change estimated by the time-series models. The median annual increase was 0.03% (IQR −3.22% to 3.54%), with estimates ranging from −16% to 26% (Figure 5). Increasing PPS frequency to quarterly improved both the precision and accuracy of the estimates (median 0.73%; IQR −0.97% to 2.22%) (Figure S5). PPS simulations of piperacillin/tazobactam demonstrated similarly wide ranges of estimates (Figure S6).

### Impact of an AMS intervention

A piecewise time variable improved the time-series models of consumption of both meropenem and piperacillin/tazobactam. The piecewise model estimated an annual increase in meropenem consumption of 1.32% (95% CI −1.01% to 3.71%) before the AMS expansion, followed by an annual reduction of 5.31% (95% CI −4.81% to 14.4%; Figure 2b). The same approach indicated a pre-intervention annual reduction in piperacillin/tazobactam consumption prior to the AMS expansion of 1.04% (95% CI −0.64% to 2.69%), which declined further by 2.39% annually (95% CI −3.72% to 8.16%) following the AMS intervention. The observed change in meropenem consumption post-intervention appeared non-linear, however, with a significant reduction in the first year being offset by an increase in the second (Figure S7).

## Discussion

Children are almost universally exposed to antibiotics in early life^13^ and the surveillance of antimicrobial consumption is a critical component of efforts to mitigate the development of AMR. In children, this surveillance is hampered by a lack of appropriate metrics and by the limitations of existing data sources to accurately quantify consumption. Our use of a rich dataset of antimicrobial administrations provides a comprehensive picture of antimicrobial use over time in our large tertiary children’s hospital setting.

We demonstrate that antimicrobial exposure in this setting is substantial, particularly in PICU. Median overall prescribing was 617 DOT/1000 patient-days and 1413 DOT/1000 patient-days in PICU. Non-PICU consumption declined over the study period by 1.77% per year (95% CI 0.50% to 3.02%). A small number of studies have utilized inpatient electronic prescribing datasets to report comparable data in children. A multicentre study from the USA produced estimates of antimicrobial consumption from 31 freestanding children’s hospitals, which are similar to our own estimates (overall consumption ∼770 DOT/1000 patient-days).^14^ A single-centre study from a children’s hospital in Canada observed higher consumption at baseline and a reduction over the course of the study from 940 to 760 DOT/1000 patient-days between 2010 and 2014.^15^ These estimates are higher than those reported from a secondary care setting in the Netherlands (230 to 450 DOT/1000 patient-days).^16^ Comparisons between institutions are challenging, being influenced by the patient population and the range of interventions undertaken in each setting. These data may suggest a range of expected consumption for children in comparable settings against which benchmarking may be possible.

The substantial variability and seasonality of prescribing observed in our study reinforce the limitations of PPSs for monitoring temporal trends in antimicrobial consumption. In contrast, the use of electronic prescribing datasets provides a valuable opportunity to evaluate antimicrobial consumption over time. We demonstrate the use of continuous data to develop robust inferences over time, confirming this as a superior approach to the use of interval data. This methodological approach also allows us to report the impact of an AMS intervention with confidence. We demonstrate that the introduction of an enhanced AMS service was associated with a subsequent 7% relative annual reduction in meropenem use and an accelerated decline of piperacillin/tazobactam use (from 1.0% to 2.4%). The initial reduction in meropenem consumption post-intervention appeared to be followed by a subsequent increase, however, demonstrating the importance of the continuous observation allowed by our analytical approach. Our analysis also reveals different patterns of prescribing during weekdays and weekends. It is not unexpected to observe a higher rate of antimicrobial prescribing for children admitted at the weekends. Important clinical and diagnostic services may be more limited at weekends, there are fewer AMS rounds and there may be less input from senior decision-makers. Our analysis revealed discordant trends in prescribing following the AMS expansion. While weekday prescribing declined, we observed an increase in prescribing at the weekends. Such insight should inform the design of appropriate AMS interventions that can address these specific patterns of behaviour.

Recent efforts to quantify consumption in children nationally and internationally have analysed large pharmacy dispensing datasets.^17^^,^^18^ Though these analyses provide substantial insight into the global picture, they offer limited information on prescribing at the patient level, and by aggregating annual data provide limited resolution to detect temporal changes in consumption. To estimate hospital consumption, we used administration data in preference to pharmacy dispensary data, but this is only possible where electronic records of drug administration are available. There is a discrepancy between the quantity of antimicrobials dispensed from the pharmacy and consumption on children’s wards, where individual doses may be reconstituted and prepared. Medicines may expire and vials are shared in sterile production units. A study of antimicrobial consumption in hospitalized children previously observed that the use of dispensary data significantly overestimated consumption compared with administration data.^19^ Interestingly, the investigators also observed that the data source was a more important source of variation than the reported metric (DDDs versus DOT), although we also observed that in most cases DDDs and DOT were highly correlated; for some antibacterials the derived models reported significantly different trends in consumption over time. It should be noted that our analysis of DDDs, derived from administration data, likely represents a more accurate picture of what patients actually received than DDDs derived from pharmacy stock control. This further strengthens our argument of DOT being the more informative metric and that while DDDs can be conveniently calculated using the electronic datasets, this metric is not useful to benchmark institutions and may provide misleading inferences regarding antimicrobial consumption over time.

We have focused our analysis on reporting trends in antimicrobial consumption. While this is important, AMS requires an understanding of the appropriateness of antimicrobial use. Considering this, we have replicated analyses published in the ECDC Annual Epidemiological Report visualizing antimicrobial use by major antimicrobial subgroup over time. We also present the breakdown over time according to the recently described England-adapted AWaRe index. In 2019, the WHO tasked governments with a target of increasing the proportion of antimicrobials prescribed from the Access group to 60% of all antimicrobial use. Such national and international recommendations may be less relevant to highly specialized institutions, where there is a clear advantage to adopting prescribing policies based on local antibiograms. Monitoring the proportion of Watch and Reserve antimicrobials prescribed in an institution over time, however, is likely to be a worthwhile objective.

The increasing availability of digital systems in healthcare affords an opportunity to explore rich datasets. In this context, the use of electronic prescribing systems offers a source of continuous data able to be translated into appropriate metrics of antimicrobial consumption in children. We believe these data offer a valuable opportunity to perform detailed temporal analysis of antimicrobial use in our institution and beyond. Our simulations of repeated PPSs of consumption of both meropenem and piperacillin/tazobactam indicate the limitations of this method. The substantial day-to-day variability makes the inference of trends by this method problematic, while the resources required to capture these data manually preclude more frequent measurement. Where electronic prescribing data exist, the focus should be on developing systems for their capture, and transformation into meaningful measures of consumption and appropriateness, which can be fed back to prescribers in the form of targeted stewardship interventions.

### Strengths and limitations

We present the findings of an analysis of antimicrobial prescribing in a large tertiary and quaternary children’s hospital. We provide data on more than 1.3 million antimicrobial prescriptions over 9 years. Our analysis includes models of overall and individual antimicrobial consumption, along with consumption grouped by antimicrobial subgroups and by the recently established AWaRe classification. Because of a later implementation of electronic prescribing on the PICU, our analysis includes only 3 years of PICU data.

This is a single-centre study, in a large tertiary care hospital with well-established electronic prescribing systems. These results therefore may not be directly comparable to less-specialist settings, or healthcare systems without electronic recording systems, where aggregated pharmacy dispensing data or PPS programmes are the most suitable alternative monitoring methodologies. Nonetheless, these results can still be used to inform the implementation of these methodologies and interpretation of their results.

The analysis focuses on inpatient prescribing alone and does not incorporate corresponding clinical data regarding the indications for antimicrobial use, or the complexity and acuity of the patient population. We have not incorporated rates of AMR, which may both influence, and be influenced by, rates of antimicrobial consumption. We do not address the difficulties of determining an acceptable, still-less-optimal level of consumption in children. This might only be offered by a multicentre study. Benchmarking overall consumption across institutions will remain complicated by the limitations of using consumption as a surrogate for quality prescribing. Measures such as the ‘standardized antimicrobial administration ratio’ developed by the US CDC and the ‘antibiotic spectrum index’ attempt to provide context for quantitative consumption data that take account of the patient population and the range of pathologies treated.^20^^,^^21^ These are interesting but as yet uncommon indices that merit further investigation.

### Conclusions

Our analysis is intended to demonstrate the value of this approach to the use of electronic prescribing data. In highly resourced settings where digital systems are established, we recommend this approach to measuring and reporting antimicrobial consumption in hospitalized children. With the caveats above, these data may be used to benchmark similar institutions against metrics of both antimicrobial consumption and appropriateness. We have demonstrated their value in inferring robust temporal trends within institutions and for testing the impact of interventions. Further evaluation might draw a clearer relationship of the contribution of these detailed consumption data on the development of AMR.

## Supplementary Material

dkab187_Supplementary_Data
