# Supplementary material for: The use of continuous electronic prescribing data to infer trends in antimicrobial consumption and estimate the impact of stewardship interventions in hospitalized children
Source: J Antimicrob Chemother. 2021 Jun 10;76(9):2464–71. doi: 10.1093/jac/dkab187 (PMC8361331; doi:10.1093/jac/dkab187)
Supplement: dkab187_Supplementary_Data [file dkab187_supplementary_data.docx]

Supplementary data

Author: Samuel Channon-Wells

Contents:

- Section 1 – Supplementary tables and figures
- Section 2 – Supplementary methods

Section 1 – Supplementary tables and figures

**Table S1:** **Correlation and estimated trends between DOT and DDD (per 1000-patient days) – all antimicrobials**

| Antimicrobial | Median DOT per 1000-pd | Pearson's r | r 95% CI | R squared | DOT estimate | DOT 95% CI | DDD estimate | DDD 95% CI |
| --- | --- | --- | --- | --- | --- | --- | --- | --- |
| Amikacin | 76.3 | 0.88 | 0.87 to 0.89 | 0.77 | -3.84 | -5.19 to -2.47 | -3.32 | -4.80 to -1.82 |
| Amoxicillin | 0 | 0.75 | 0.73 to 0.78 | 0.57 | -1.79 | -3.66 to 0.11 | -1.15 | -2.66 to 0.39 |
| Ampicillin | 0 | 0.65 | 0.61 to 0.70 | 0.42 | -3.59 | -5.4 to -1.75 | -2.79 | -4.16 to -1.39 |
| Azithromycin | 19.3 | 0.88 | 0.87 to 0.89 | 0.77 | 3.86 | 1.45 to 6.33 | 1.64 | -0.55 to 3.88 |
| Aztreonam | 0 | 0.91 | 0.89 to 0.93 | 0.83 | -0.55 | -2.07 to 0.99 | -0.47 | -2.03 to 1.11 |
| Benzylpenicillin | 8.5 | 0.57 | 0.54 to 0.59 | 0.32 | -8.50 | -10.12 to -6.86 | -4.11 | -5.89 to -2.29 |
| Cefalexin | 0 | 0.71 | 0.67 to 0.76 | 0.51 | -1.96 | -3.89 to 0.00 | -2.13 | -3.38 to -0.86 |
| Cefixime | 0 | 0.88 | 0.83 to 0.92 | 0.77 | -1.78 | -3.20 to -0.34 | -2.08 | -3.34 to -0.81 |
| Cefotaxime | 5.3 | 0.67 | 0.64 to 0.70 | 0.45 | -4.33 | -6.51 to -2.10 | -3.01 | -5.25 to -0.73 |
| Cefoxitin | 0 | 0.93 | 0.92 to 0.94 | 0.86 | -0.56 | -3.03 to 1.97 | -1.36 | -2.86 to 0.17 |
| Cefradine | 0 | 0.56 | 0.51 to 0.63 | 0.31 | -2.54 | -3.80 to -1.27 | -2.40 | -3.65 to -1.14 |
| Ceftazidime | 15.3 | 0.84 | 0.83 to 0.85 | 0.71 | -1.08 | -3.23 to 1.11 | -0.30 | -2.65 to 2.12 |
| Ceftolozane/Tazobactam | 0 | 0.99 | 0.96 to 1.00 | 0.97 | -1.88 | -3.14 to -0.61 | -1.79 | -3.06 to -0.52 |
| Ceftriaxone | 3.7 | 0.83 | 0.82 to 0.85 | 0.70 | 0.73 | -1.59 to 3.10 | 0.98 | -1.21 to 3.21 |
| Cefuroxime | 0 | 0.75 | 0.73 to 0.77 | 0.57 | -4.32 | -6.15 to -2.47 | -3.54 | -5.23 to -1.82 |
| Chloramphenicol | 0 | 0.96 | 0.90 to 1.00 | 0.93 | -2.26 | -3.51 to -1.00 | -2.25 | -3.49 to -0.99 |
| Ciprofloxacin | 55.1 | 0.85 | 0.84 to 0.86 | 0.73 | -0.63 | -2.36 to 1.14 | 2.43 | 0.54 to 4.35 |
| Clarithromycin | 13.5 | 0.78 | 0.76 to 0.80 | 0.61 | -1.14 | -3.82 to 1.62 | -0.86 | -3.07 to 1.41 |
| Clindamycin | 5.4 | 0.74 | 0.72 to 0.76 | 0.55 | -1.38 | -4.02 to 1.32 | -1.55 | -3.67 to 0.62 |
| Clofazimine | 0 | 0.95 | 0.94 to 0.96 | 0.90 | -1.44 | -2.88 to 0.03 | -1.60 | -3.04 to -0.15 |
| Co-amoxiclav | 74.8 | 0.54 | 0.51 to 0.56 | 0.29 | -0.63 | -1.98 to 0.74 | -0.18 | -1.90 to 1.56 |
| Colistin | 2.6 | 0.88 | 0.86 to 0.89 | 0.77 | -1.41 | -3.91 to 1.15 | -1.74 | -3.31 to -0.15 |
| Co-trimoxazole | 25.1 | 0.94 | 0.94 to 0.95 | 0.89 | -0.72 | -2.59 to 1.19 | -1.33 | -3.36 to 0.74 |
| Cycloserine | 0 | 0.87 | 0.86 to 0.88 | 0.75 | -0.35 | -2.92 to 2.29 | -1.88 | -3.13 to -0.60 |
| Dapsone | 0 | 0.87 | 0.86 to 0.89 | 0.77 | -2.43 | -4.10 to -0.72 | -2.71 | -4.54 to -0.85 |
| Daptomycin | 0 | 0.94 | 0.92 to 0.96 | 0.88 | -1.77 | -3.23 to -0.30 | -1.91 | -3.17 to -0.64 |
| Doxycycline | 0 | 0.89 | 0.88 to 0.91 | 0.79 | -1.95 | -3.56 to -0.31 | -1.96 | -3.63 to -0.27 |
| Ertapenem | 0 | 0.93 | 0.89 to 0.97 | 0.86 | -2.05 | -3.31 to -0.78 | -2.03 | -3.29 to -0.76 |
| Erythromycin | 5 | 0.58 | 0.55 to 0.61 | 0.34 | -4.26 | -6.92 to -1.51 | -4.83 | -6.52 to -3.11 |
| Ethambutol | 4.1 | 0.70 | 0.68 to 0.72 | 0.49 | -0.30 | -4.43 to 4.00 | 0.46 | -1.92 to 2.90 |
| Flucloxacillin | 33 | 0.77 | 0.75 to 0.79 | 0.59 | -3.92 | -5.34 to -2.48 | -2.60 | -4.35 to -0.83 |
| Fusidic Acid | 0 | 0.86 | 0.83 to 0.88 | 0.74 | -2.79 | -4.18 to -1.38 | -2.53 | -3.78 to -1.26 |
| Gentamicin | 0 | 0.77 | 0.74 to 0.79 | 0.59 | -4.67 | -7.01 to -2.27 | -4.14 | -5.99 to -2.25 |
| Imipenem | 0 | 0.89 | 0.88 to 0.90 | 0.79 | 5.06 | 1.89 to 8.34 | 6.19 | 3.09 to 9.38 |
| Isoniazid | 7.7 | 0.83 | 0.81 to 0.84 | 0.69 | -9.60 | -14.39 to -4.55 | -3.52 | -7.38 to 0.51 |
| Levofloxacin | 0 | 0.96 | 0.94 to 0.99 | 0.93 | -2.13 | -3.38 to -0.87 | -2.14 | -3.39 to -0.88 |
| Linezolid | 3.3 | 0.83 | 0.82 to 0.84 | 0.69 | 5.10 | 2.43 to 7.83 | 1.19 | -0.91 to 3.33 |
| Meropenem | 35.5 | 0.82 | 0.81 to 0.83 | 0.67 | 0.45 | -1.48 to 2.43 | 0.68 | -1.40 to 2.81 |
| Metronidazole | 19.2 | 0.80 | 0.78 to 0.82 | 0.64 | -6.05 | -7.86 to -4.20 | -3.42 | -5.22 to -1.59 |
| Minocycline | 0 | 0.90 | 0.88 to 0.92 | 0.81 | -3.53 | -5.21 to -1.82 | -2.74 | -4.26 to -1.19 |
| Moxifloxacin | 2.3 | 0.92 | 0.91 to 0.93 | 0.85 | 12.12 | 8.38 to 15.99 | 7.02 | 4.43 to 9.67 |
| Nalidixic Acid | 0 | 1.00 | 1.00 to 1.00 | 1.00 | -2.29 | -3.54 to -1.03 | -2.25 | -3.50 to -0.99 |
| Nitrofurantoin | 2.5 | 0.63 | 0.60 to 0.66 | 0.39 | 4.36 | 2.06 to 6.70 | -1.41 | -2.80 to -0.01 |
| Ofloxacin | 0 | 0.96 | 0.94 to 0.98 | 0.92 | -2.03 | -3.28 to -0.76 | -2.12 | -3.36 to -0.85 |
| Penicillin V | 19.1 | 0.81 | 0.79 to 0.82 | 0.66 | -4.75 | -6.53 to -2.93 | -1.90 | -3.44 to -0.34 |
| Piperacillin/Tazobactam | 65.1 | 0.90 | 0.89 to 0.90 | 0.81 | -1.21 | -2.69 to 0.30 | -1.02 | -2.63 to 0.61 |
| Pivmecillinam | 0 | 0.70 | 0.65 to 0.77 | 0.50 | -1.12 | -2.40 to 0.17 | -1.67 | -2.93 to -0.39 |
| Protionamide | 0 | 0.89 | 0.89 to 0.91 | 0.80 | -1.76 | -3.95 to 0.48 | -1.96 | -3.53 to -0.36 |
| Pyrazinamide | 0 | 0.89 | 0.88 to 0.90 | 0.79 | -3.29 | -5.44 to -1.09 | -2.83 | -5.94 to 0.39 |
| Rifampicin | 8.3 | 0.69 | 0.67 to 0.71 | 0.48 | -10.24 | -13.15 to -7.23 | -4.95 | -7.59 to -2.24 |
| Sulfadiazine | 0 | 0.86 | 0.82 to 0.89 | 0.74 | -1.55 | -3.06 to -0.03 | -1.09 | -3.29 to 1.16 |
| Teicoplanin | 22.8 | 0.87 | 0.86 to 0.88 | 0.76 | -1.87 | -3.84 to 0.14 | -1.04 | -2.83 to 0.78 |
| Tetracycline | 0 | 0.97 | 0.95 to 0.99 | 0.94 | -2.24 | -3.69 to -0.77 | -2.27 | -3.52 to -1.00 |
| Ticarcillin/Clavulanate | 0 | 0.80 | 0.75 to 0.85 | 0.64 | -3.18 | -4.66 to -1.68 | -2.68 | -3.93 to -1.42 |
| Tigecycline | 2.5 | 0.91 | 0.90 to 0.92 | 0.83 | -0.11 | -2.94 to 2.80 | -0.34 | -2.76 to 2.13 |
| Tobramycin | 9.7 | 0.88 | 0.87 to 0.89 | 0.78 | 9.21 | 6.72 to 11.77 | 10.9 | 8.05 to 13.83 |
| Trimethoprim | 12.4 | 0.55 | 0.52 to 0.57 | 0.30 | -0.93 | -3.30 to 1.50 | -0.62 | -2.10 to 0.88 |
| Vancomycin | 27.8 | 0.84 | 0.83 to 0.86 | 0.71 | -6.06 | -7.80 to -4.28 | -4.57 | -6.46 to -2.65 |

**Table S2: England-adapted AWaRe index prescribing**

| Year | Access | Watch | Reserve | Other |
| --- | --- | --- | --- | --- |
| 2010 | 165.3 (24.7) | 430.2 (64.4) | 57.9 (8.7) | 14.8 (2.2) |
| 2011 | 158.3 (24.1) | 425.6 (64.7) | 58.9 (9.0) | 15.1 (2.3) |
| 2012 | 154.7 (23.5) | 421.8 (64.0) | 65.8 (10.0) | 16.4 (2.5) |
| 2013 | 146.4 (23.1) | 415.9 (65.6) | 57.7 (9.1) | 14.2 (2.2) |
| 2014 | 149.8 (21.8) | 441.6 (64.3) | 65.6 (9.6) | 29.5 (4.3) |
| 2015 | 129.2 (21.1) | 402.1 (65.7) | 65.3 (10.7) | 15.9 (2.6) |
| 2016 | 133.8 (21.8) | 402.2 (65.7) | 66.5 (10.9) | 10.1 (1.6) |
| 2017 | 117.3 (20.1) | 394.0 (67.7) | 58.9 (10.1) | 12.1 (2.1) |
| 2018 | 128.2 (22.0) | 375.4 (64.5) | 68.5 (11.8) | 9.5 (1.6) |

Table S2: Days-On-Therapy (DOT) per 1000 patient-days for each antimicrobial class from the England-adapted AWaRe index during each year of the study. Number inside brackets represents percentage of antimicrobial use of each class per year, as measured by DOT

**Figure S1: Prescribing trends by weekday**


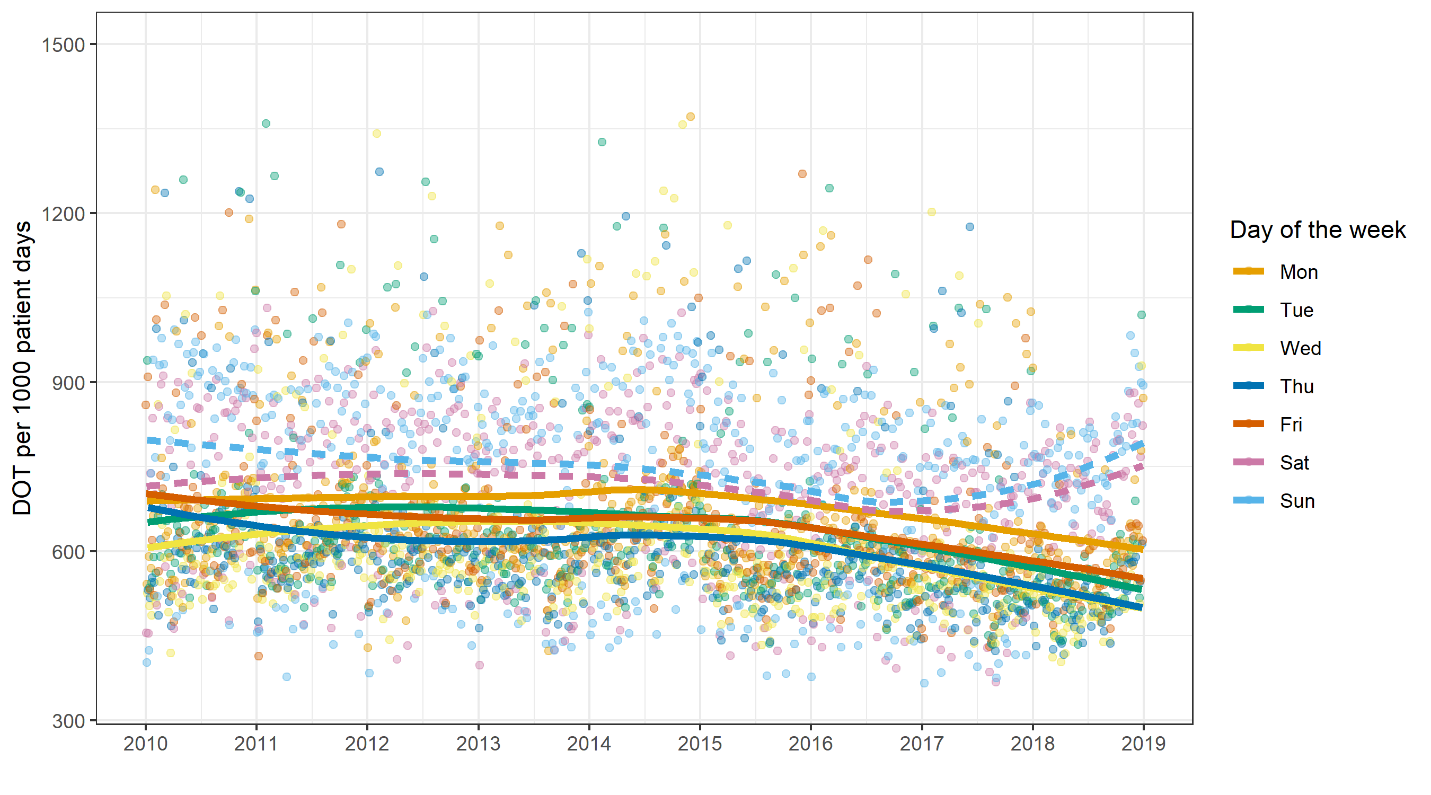


Figure S1: DOT per 1000 patient days of all antimicrobials prescribed, separated by day of the week. Smoothed lines fitted with loess regression. Dashed lines represent weekend days

**Figure S2: Prescribing trends by weekend classification**


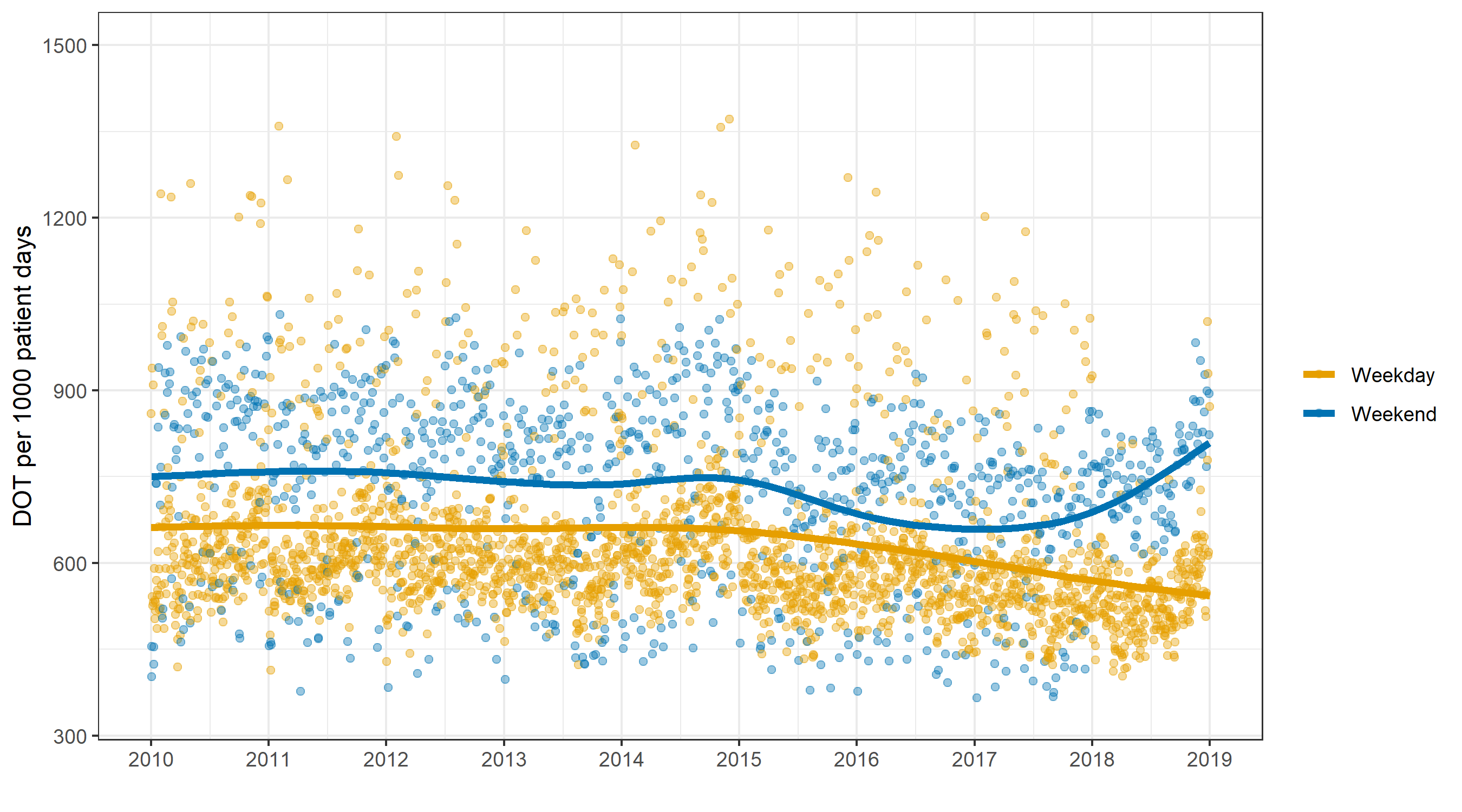


Figure S2: Average DOT per 1000 patient days of all antimicrobials prescribed, summarised weekly

**Figure S3: Piperacillin/Tazobactam DOT and DDD models**


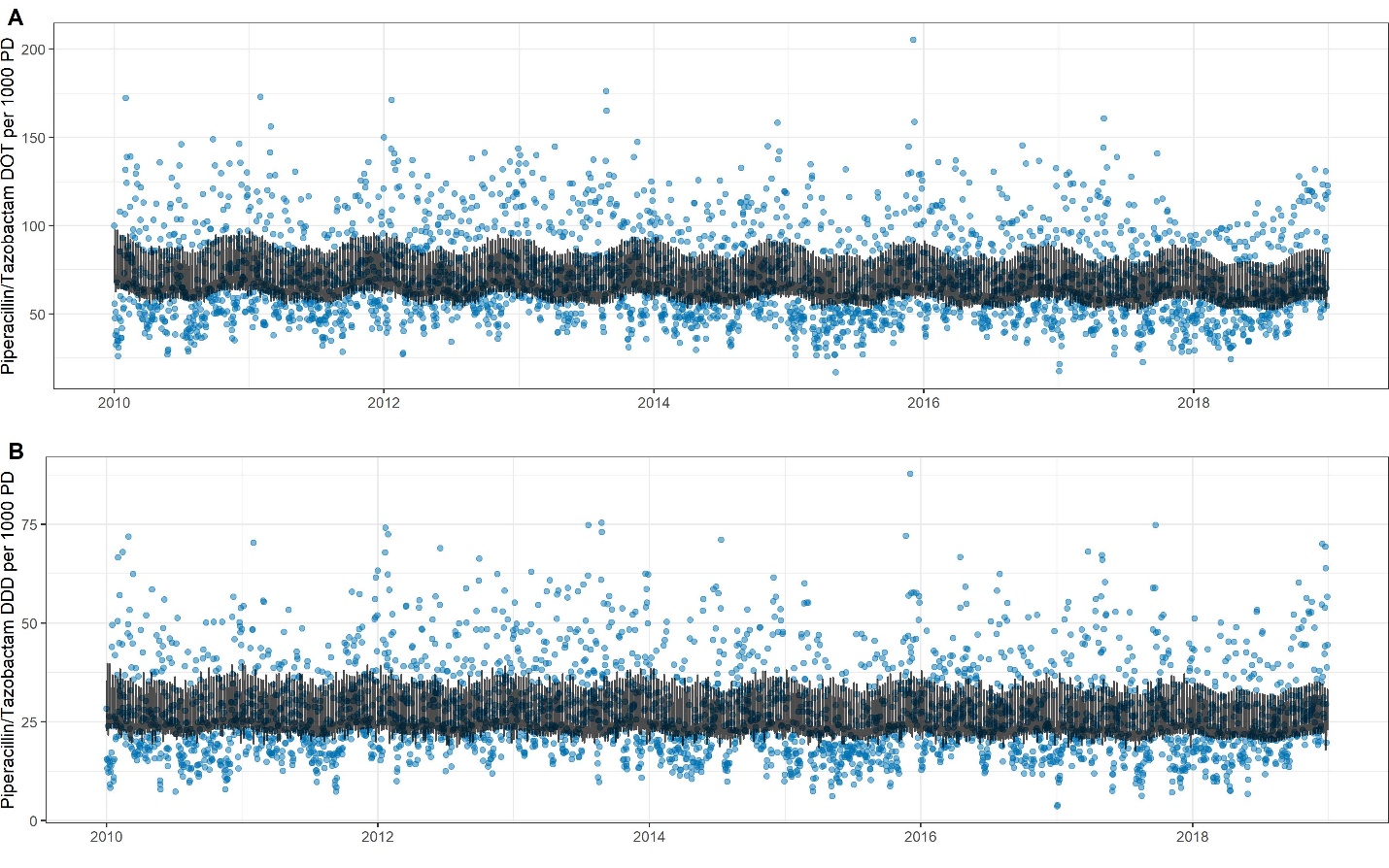


Figure S3: A) Piperacillin/tazobactam DOT per 1000-patient-days, B) Piperacillin/tazobactam DDD per 1000-patient-days

**Figure S4: Ciprofloxacin dose change by year**


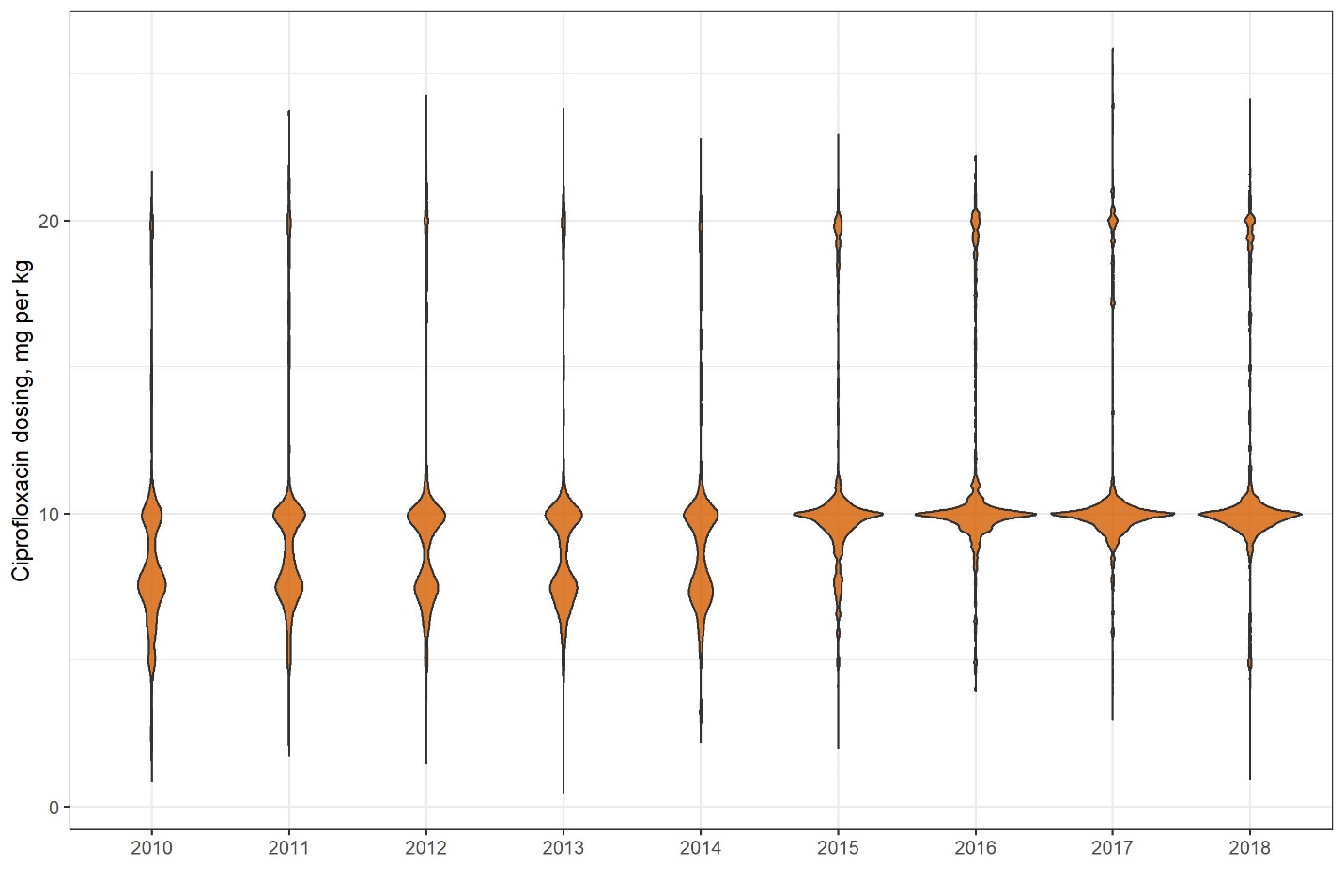


Figure S4: Violin plot of ciprofloxacin doses in mg/kg in each year from 2010 to 2018, demonstrating reduction in doses at 7.5mg/kg and an increase in doses at 10mg/kg in 2015

**Figure S5: Quarterly Meropenem PPS simulations**


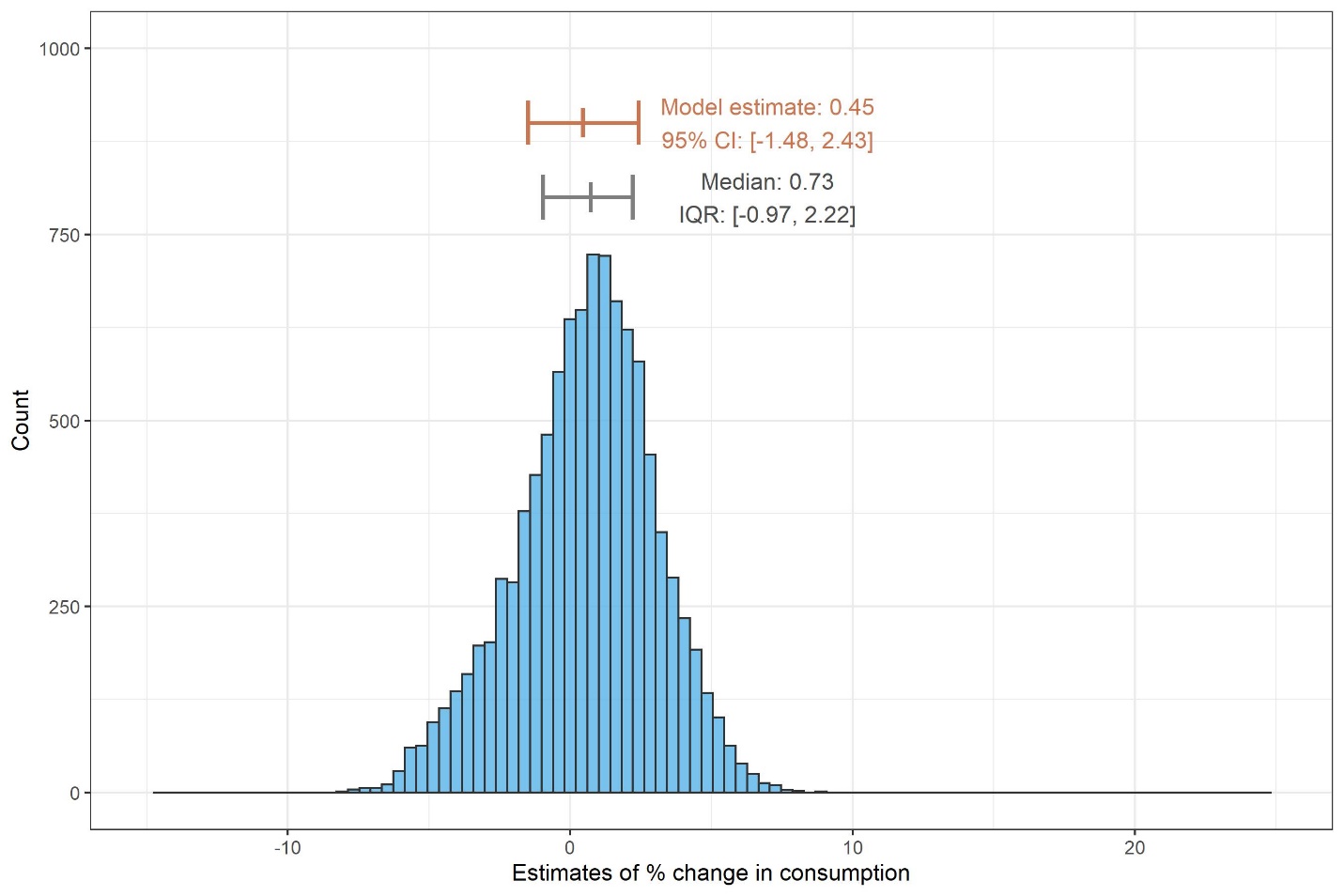


Figure S5: Frequency distribution of 10000 simulations of the annual change in meropenem consumption estimated by a quarterly PPS

**Figure S6: Annual Piperacillin/Tazobactam PPS simulations**


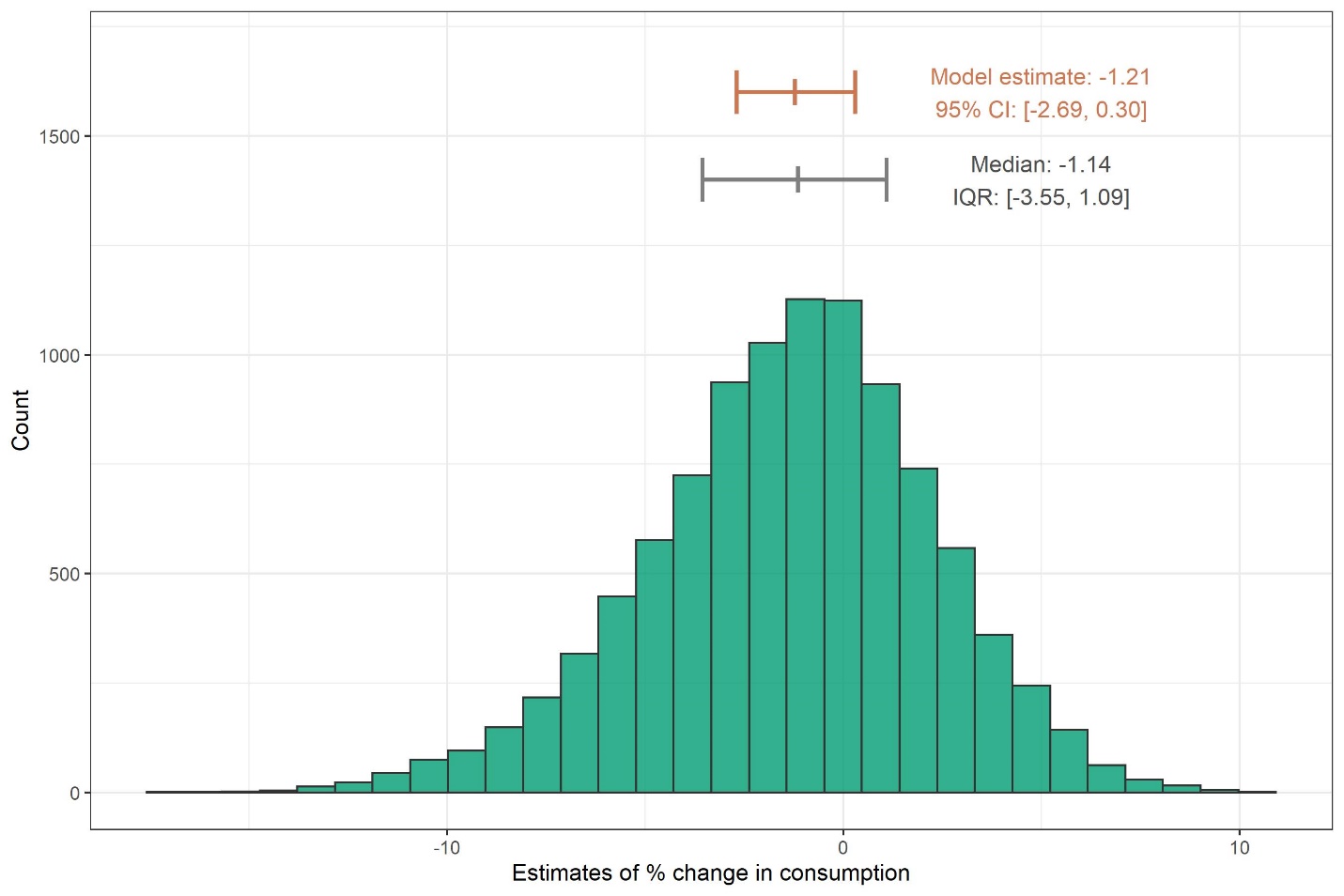


Figure S6: Frequency distribution of 10000 simulations of the annual change in piperacillin/tazobactam consumption estimated by a yearly PPS

**Figure S7: Meropenem additional split model**


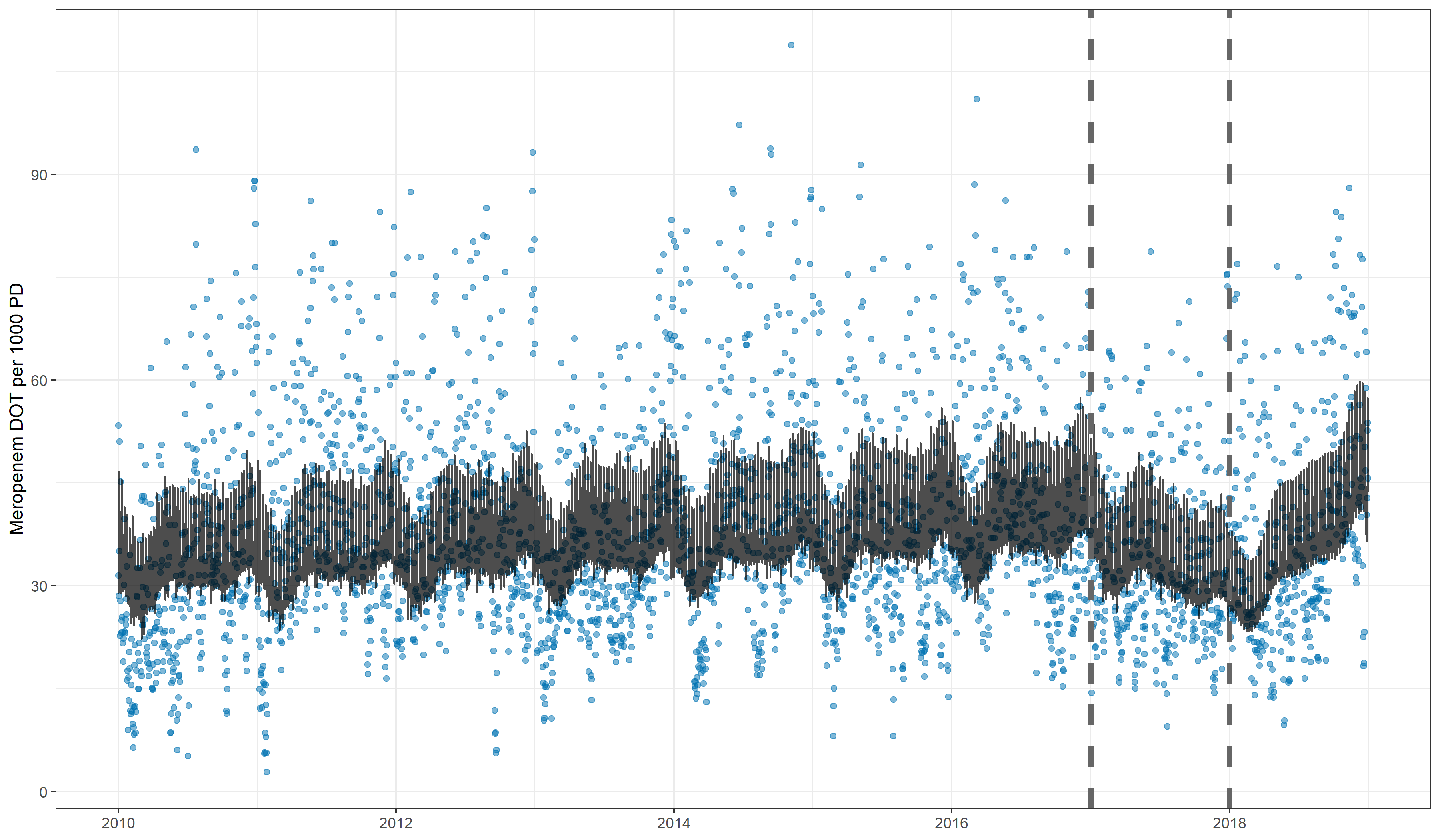


Figure S7: Meropenem consumption (DOT per 1000 patient-days), using an autoregressive moving-average piecewise fit, with splits at the time of the expansion of AMS services at the end of 2016, and one year later (dashed vertical lines)

Section 2 – Supplementary methods

**Section 2.1 - Handling missing values**

Drug administration data on almost all antimicrobials were missing from 3 dates and there were extremely low outlier values of DOT & DDD per 1000-patient days (rate) on dates immediately surrounding the missing dates. When we could reasonably assume the outlier values to be incorrect, we estimated the true rate using the median of all other values. Rates from missing dates were imputed using the overall median. We ran sensitivity analyses using the global mean as an estimate for missing and outlier values, and using local averages 3-days either side of missing values. This confirmed that the method used did not significantly alter results of any statistical analyses.

**Section 2.2 - Time series models:**

Scatter plots and generalised additive model plots were inspected to look for evidence of non-linearity on the log-odds scale.

DOT & DDD were first subjected to a log-transform, which improved the approximation to the normality assumption. Both metrics were then modelled using an autoregressive moving-average model, with autoregressive order 1 and moving-average order 1. This was implemented in R using the arima0 function. We modeled seasonality by including sine-cosine variables with periods of 12 months, 6 months, and 4 months. A seven-level factor for the day of the week was used to correct for variations in prescribing by weekday. Finally, patient days (log-transformed) were used as an offset in the model.

This model is implemented below:

data <- read.csv("raw_data/nonicu_data/nonicu_all_DOT.csv")
data$date <- as.Date(data$date)
data <- data[order(data$date),]
n <- dim(data)[1]

# Days-on-therapy variable:
dot <- data$n

# Time variable:
time <- 1:n

# Generalised additive model plots to look for evidence of non-linearity:
gam.DOT <- gam(dot ~ s(time), method = "REML")
plot(gam.DOT, xlab = "time (days)", ylab = "DOTs", main = "GAM, All antimicrobials (test for non-linearity)")


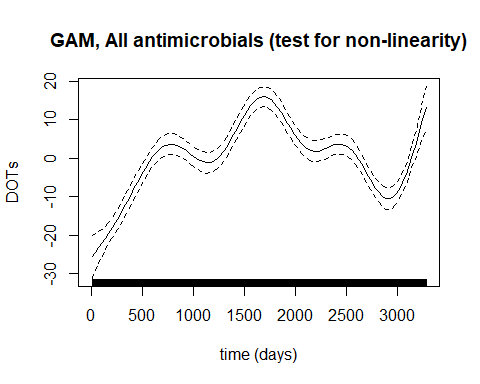


# Cosine-sine variables to model seasonality:
ss <- sin(2*pi*time/365) # 12 month period
cc <- cos(2*pi*time/365) # 12 month period
ss2 <- sin(2*pi*2*time/365) # 6 month period
cc2 <- cos(2*pi*2*time/365) # 6 month period
ss3 <- sin(2*pi*3*time/365) # 4 month period
cc3 <- cos(2*pi*3*time/365) # 4 month period

# Day of the week factor variable:
days <- as.factor(lubridate::wday(data$date))

# Patient days variable, used as an offset:
pd <- data$pd

# Reconfigure data for use in arima0 function:
Xmat <- model.matrix(~ -1 + days + time + cc + ss + cc2 + ss2 + cc3 + ss3)
# remove first column because the arima fitting function automatically adds a column for an intercept
Xmatrix <- Xmat[,-1]
# trick the arima fitting function to include the offset
Yvariable <- log(dot+1)-log(pd)

# fit model:
fit_arma1.1 <- arima0(Yvariable,order=c(1,0,1),xreg = Xmatrix) # fit ARMA(1,1) autocorrelation

Fitted values were plotted against residuals to detect any underlying relationship.


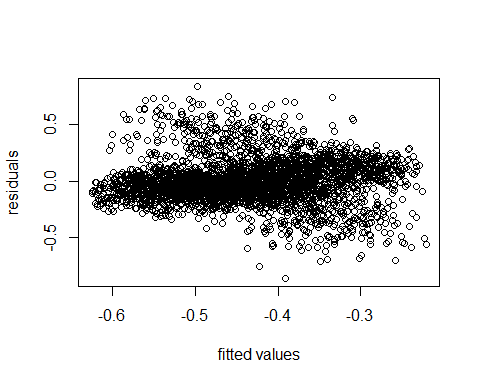


Plots of autocorrelation of residuals were also inspected for abnormalities


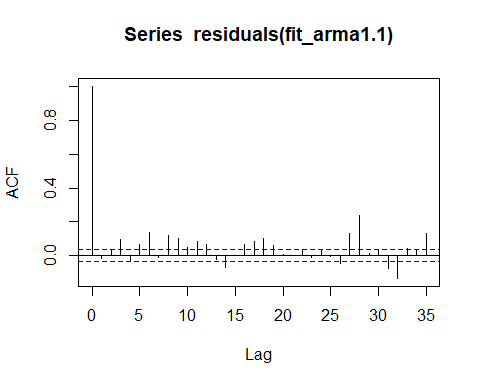


The time coefficient was used to estimate the yearly percentage change in consumption, with standard errors used to calculate the upper and lower confidence interval bounds.

## estimates lower_ci upper_ci
## time -1.771979 -3.024071 -0.5037201

**Section 2.3 - Split time-series models (meropenem):**

A piecewise autoregressive moving-average (ARMA) model, with breakpoint specified by the date of the AMS intervention (the end of 2016) was used to examine trends before and after the AMS expansion. We then estimated yearly percentage change in consumption both before and after the AMS expansion. Example code from meropenem piecewise ARMA modelling is displayed below:

# Load and format data:
data <- read.csv("raw_data/nonicu_data/nonicu_meropenem_DOT.csv")
data$date <- as.Date(data$date)
data <- data[order(data$date),]
# Days-on-therapy variable:
dot <- data$n

# New time variables:
time1 <- time
time2 <- ifelse(time > 2557, time - 2557, 0)

# Reconfigure data for use in arima0 function:
Xmat <- model.matrix(~ -1 + days + time1 + time2 + cc + ss + cc2 + ss2 + cc3 + ss3)
# remove first column because the arima fitting function automatically adds a column for an intercept
Xmatrix <- Xmat[,-1]
# trick the arima fitting function to include the offset
Yvariable <- log(dot+1)-log(pd)

# fit model:
fit_arma1.1 <- arima0(Yvariable,order=c(1,0,1),xreg = Xmatrix) # fit ARMA(1,1) autocorrelation

# obtain fitted values
N <- dim(Xmatrix)[1]; one<-rep(1,N)
fv_arma1_1 <- cbind(one,Xmatrix) %*% fit_arma1.1$coef[-c(1,2)]

# Residuals vs fitted value plot:
plot(fv_arma1_1, residuals(fit_arma1.1), xlab = "fitted values", ylab = "residuals") # and residual plot looks nicer than before!


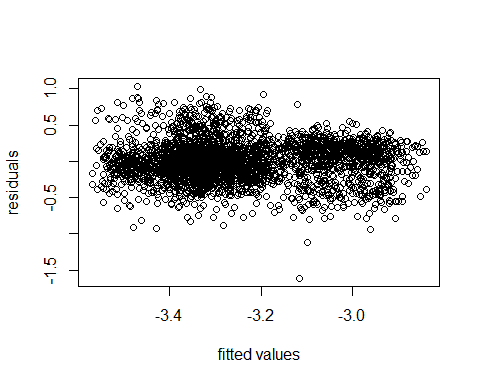


# Autocorrelation plot of Pearson residuals:
acf(residuals(fit_arma1.1))


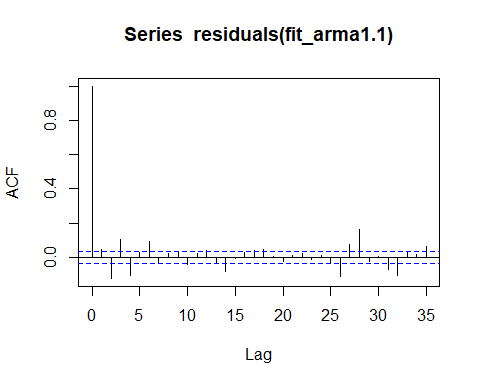


# obtain estimates of percentage change
results_arma1_1 <- cbind(fit_arma1.1$coef[-c(1,2)],sqrt(diag(fit_arma1.1$var.coef))[-c(1, 2)])
results_arma1_1 <- as.data.frame(cbind(results_arma1_1)); colnames(results_arma1_1)[1:2] <- c("estimates", "se")
results_arma1_1$lower_ci <- results_arma1_1$estimates + qnorm(0.025)*results_arma1_1$se
results_arma1_1$upper_ci <- results_arma1_1$estimates + qnorm(0.975)*results_arma1_1$se

ests <- results_arma1_1[c("time1", "time2"), c(1, 3, 4)]
(exp(ests)^365 - 1)*100

## estimates lower_ci upper_ci
## time1 1.321370 -1.012025 3.709770
## time2 -6.635078 -15.765532 3.485056

**Section 2.4 - Comparison of DOT and DOT metrics:**

We performed two comparisons of DOT and DDD metrics for each antimicrobial:

- Direct comparison of DOT and DD metrics using a linear model with intercept, reporting $R^{2}$ for this model, and the Pearson Product-Moment Correlation Coefficient
- Comparison of the estimated yearly change in consumption using each metric, using the above ARMA model

**Section 2.5 - Point-prevalence survey (PPS) simulations:**

To conduct yearly PPS simulations we extracted DOT data for a single day in each year of the study. The first day was randomly selected from all weekdays in 2010. Subsequent days were selected one year on, plus random noise (discrete uniform random noise added to each day (range +/- 4 days)). If this fell on a weekend, we randomly selected either the preceding Thursday/Friday or following Monday/Tuesday.

For each simulation, we used the data collected from these 9 days to estimate a percentage yearly change in consumption, using a log-transformed linear model. We report the median, IQR, and range of these estimates generated from simulations, and plot the estimates in histograms to show their distribution.

Sensitivity analyses altering the level of random noise and/or allowing weekend sampling showed that these alternative cases generated essentially the same results.

The analogous method was used for simulating quarterly PPS.
